# Chemical-neuroanatomical organization of peripheral sensory-efferent systems in the pond snail (*Lymnaea stagnalis*)

**DOI:** 10.1007/s00429-020-02145-z

**Published:** 2020-09-20

**Authors:** Réka Horváth, Izabella Battonyai, Gábor Maász, János Schmidt, Zsuzsanna N. Fekete, Károly Elekes

**Affiliations:** 1 Department of Experimental Zoology, Centre for Ecological Research, Balaton Limnological Institute, 8237 Tihany, Hungary; 2 Department of Analytical Biochemistry, Institute of Biochemistry and Medical Chemistry, University of Pécs, 7624 Pécs, Hungary

**Keywords:** Sensory neurons, Periphery, Monoamines, Neuropeptides, Extrinsic modulation, *Lymnaea stagnalis*

## Abstract

Perception and processing of chemical cues are crucial for aquatic gastropods, for proper elaboration of adaptive behavior. The pond snail, *Lymnaea stagnalis*, is a model species of invertebrate neurobiology, in which peripheral sensory neurons with different morphology and transmitter content have partly been described, but we have little knowledge regarding their functional morphological organization, including their possible peripheral intercellular connections and networks. Therefore the aim of our study was to characterize the sensory system of the tentacles and the lip, as primary sensory regions, and the anterior foot of *Lymnaea* with special attention to the transmitter content of the sensory neurons, and their relationship to extrinsic elements of the central nervous system. Numerous bipolar sensory cells were demonstrated in the epithelial layer of the peripheral organs, displaying immunoreactivity to antibodies raised against tyrosine hydroxylase, histamine, glutamate and two molluscan type oligopeptides, FMRFamide and Mytilus inhibitory peptide. A subepithelial plexus was formed by extrinsic serotonin and FMRFamide immunoreactive fibers, whereas in deeper regions axon processess of different origin with various immunoreactivities formed networks, too. HPLC–MS assay confirmed the presence of the low molecular weight signal molecules in the three examined areas. Following double-labeling immunohistochemistry, close arrangements were observed, formed by sensory neurons and extrinsic serotonergic (and FMRFamidergic) fibers at axo-dendritic, axo-somatic and axo-axonic levels. Our results suggest the involvement of a much wider repertoire of signal molecules in peripheral sensory processes of *Lymnaea*, which can locally be modified by central input, hence influencing directly the responses to environmental cues.

## Introduction

The capturing and subsequent interpretation of external signals from the surroundings are pivotal for optimal adaptation in the animal kingdom, including invertebrates. After arthropods, mollusks are the second most important phylum among invertebrates, represented by about hundred thousand different species, in which the largest class, gastropods involve a number of species, such as the opistobranchs *Aplysia californica* and *Tritonia diomedea* or the pulmonates *Lymnaea stagnalis*, *Helix pomatia* and *Limax maximus*, which are well-known model animals for neuroscience research (Chase 2002). These species possess well-developed sensory organs, primarily the tentacles (rhinophores) and lip, although other anatomical regions such as the mantle edge and the body surface are also supplied with sensory elements (Chase 2001). These organs are set for chemo- and/or mechanosensation. In addition, aquatic pulmonate species possess a specific chemosensory organ, the osphradium, whereas the statocysts serve in all gasptropod species for gravireception. Because of their poor visual perception, the role of chemoreception in collecting even distant information from the surrounding is essential. Most of the sensory elements responsible for catching chemical cues, and so playing an initiative role in elaborating different behaviors like food-finding, foraging, mating, escape or avoidance, are concentrated in the tentacles and lip.

There are a number of reports dealing with peripheral information processing by sensory neurons located in the gastropod central nervous system (CNS) (Kandel 1976, 1979; Chase 2001). Furthermore, early and recent neuroanatomical studies including ultrastuctural investigations have described the presence, distribution, morphology and organization of sensory cells in the periphery of several gastropod species. In *Helix*, bipolar sensory neurons were visualized in the epithelial and subepithelial layers of the anterior tentacles, lips and foot (Hernádi and Benedeczky 1978, 1994; Hernádi 1981, 1982). Zaitseva and Bocharova (1981) classified six different types of sensory neurons in the head regions of *Helix* and *Viviparus*, based on their location, anatomy, presence and number of cilia and microvilli, and their central projections. In the tentacles of *Limax*, Ito et al. (2000) identified three sensory neuron subtypes, namely the round, the spindle-shaped and the small ones. In terrestrial snails (e. g. *Helix*, *Achatina*) and *Limax,* sensory cells were shown to project mainly to the tentacular ganglion, then entered to the procerebrum, meanwhile a small part of them reached directly the CNS (Chase 2001, 2002; Chase and Tolloczko 1993; Ierusalimsky and Balaban 2010). Nevertheless, the organization of the rhinophores in *Aplysia* was found to be slightly different. The bipolar sensory cells projected first to olfactory glomeruli located beneath the sensory epithelium, which were connected thereafter to the rhinophore ganglion (Wertz et al. 2006).

The peripheral nervous system of the pond snail *Lymnaea stagnalis* has also been studied in details, regarding the types of sensory neurons, the cellular organization of the sensory system and the possible role of the peripheral structures in forwarding information to the CNS. Several types of sensory dendrites were distinguished, depending on the presence or lack, as well as the number and position of cilia (Zylstra 1972a, b; Roubos and Van der Wal Divendal 1982; Dorsett 1986; Chase 2002). Recently, four different types of ciliated bipolar sensory neurons were distinguished by Wyeth and Croll (2011) in the cephalic sensory region containing the lip and tentacles, based on the position and form of the sensory dendrites, the clustering of the cell bodies, and presence or lack of the sensory axon. By applying histo- and immunohistochemical methods, dopamine (DA/tyrosine hydroxylase [TH]), histamine (HA), glutamate (Glu), nitric oxide (nitrogen monoxide synthase ([NOS]/dihydronicotinamide adenine dinucleotide phosphate diaphorase [NADPHd]) and the oligopeptide FMRFa (Fa) were demonstrated in the sensory neurons of different peripheral regions of gastropods, including the cephalic sensory organs (tentacles, lip), the foot and the mantle (TH/DA: *Lymnaea*, Voronezhskaya et al. 1999; Croll et al. 1999; Wyeth and Croll 2011; *Aplysia*, Croll 2001; *Phestilla,* Croll et al. 2003; *Pleurobranchea*, Faller et al. 2008; Brown et al. 2018; *Biomphalaria*, Vallejo et al. 2014; HA: *Lymnaea*, *Helix*, Hegedűs et al. 2004; *Lymnaea*, Wyeth and Croll 2011; *Biomphalaria*, Habib et al. 2015; Glu: *Lymnaea*, Hatakeyama et al. 2007; NOS/NADPHd: *Lymnaea*, Elphick et al. 1995; Serfőző et al. 1998; Wyeth and Croll 2011; *Aplysia*, Moroz 2006; Fa: *Limax*, Suzuki et al. 1997; *Aplysia*, Wollesen et al. 2007). The possible transmitter content was also correlated with the morphology of the sensory cells in the cephalic sensory organs of *Lymnaea* (Wyeth and Croll 2011).

Although the sensory cell types and a good part of their neurotransmitter content have been described in the cephalic region of gastropods, including *Lymnaea* (see above), the spatial organization and the functional–anatomical relationship between neurochemically different peripheral sensory structures and efferent elements originating from the CNS has not yet been investigated. Therefore, the aim of our present study was, first, to perform a detailed chemical-neuroanatomical analysis following both single and double-labeling immunohistochemistry to widen our knowledge on the presence of signaling molecules in sensory cells of the pond snail, *Lymnaea stagnalis*. In the course of this, aminergic (DA, HA), amino acidergic (Glu) and peptidergic (Fa, MIP) afferent components were visualized in the sensory epithelium. Next, the possible functional anatomical relationship of the sensory elements to other neuronal components of extrinsic (central) origin was analyzed, focusing on the serotonin (5-HT) immunoreactive (5-HT-IR) elements. 5-HT is perhaps the most studied signaling molecule in the gastropod nervous system (Walker 1986; Walker et al. 2009; Gillette 2006). 5-HT containing processes have been regarded as a major extrinsic component of central origin in the gastropod periphery, which were identified in both somatic and visceral regions. As to the the innervation of the cephalic area, the 5-HTergic cerebral giant neuron was shown to play a key role (Pentreath and Cottrell 1974; Gillette 1991; Chase 2002). The role of 5-HTergic pedal neurons was also demonstrated in the innervation of the foot, including the ciliary movement of *Lymnaea* (Syed et al. 1988; Syed and Winlow 1989; McKenzie et al. 1998). The presence of 5-HT in peripheral nerve cells was only demonstrated in transient apical (sensory) cells of gastropod embryos (Marois and Croll 1992; Kempf et al. 1997; Marois and Carew 1997; Page and Parries 2000; Voronezhskaya et al. 2004). Our investigations were also coupled with HPLC–MS assay, to quantify and support the presence of small molecular weight neurotransmitters (5-HT, DA, HA, Glu) in the cephalic organs.

## Materials and methods

### Animals

Adult specimens of the pond snail*, Lymnaea stagnalis* were used for our experiments. The animals were collected from the Kis-Balaton reservoir and other inlets, then maintained in aquaria supplied with oxygenated Balaton-water under 16–8 h light–dark cycle at room temperature (~ 16–20 °C) and fed on lettuce ad libitum.

All procedures were performed according to the protocols approved by the Scientific Committee of Animal Experimentation of the Balaton Limnological Institute (VE-I-001/0189010/2013).

### Light-microscopic immunohistochemistry

#### Fixation

The tentacles, the lip and the edge of anterior foot region were dissected from altogether 50 adult specimens. Tissues used for anti-5-HT, -HA, -Glu, -TH, -MIP and -Fa immunohistochemistry (IHC), respectively, were fixed in 4% paraformaldehyde (PFA, Reanal, Budapest) dissolved in 0.1 M phosphate buffer (PB) overnight at 4 °C. For anti-HA IHC the peripheral tissues were fixed first in a mixture of 4% 1-ethyl-3(3-dimethylaminopropyl)-carbodiimide (EDAC, Sigma) and 0.4% *N*-hydroxysuccinimide (NHS, Sigma) for 4 h at 4 °C, followed by a fixation in 2% PFA for 4 h at 4 °C. The fixatives were diluted in PB. After fixations, the tissues were washed repeatedly in 0.1 M phosphate buffered saline (PBS, pH 7.4).

#### Immunohistochemical procedure

Sixteen µm thick serial sections were cut on a cryostat (Leica Jung 1800) and mounted on chromalum-gelatine-coated slides. The immunolabeling was accomplished by two-step indirect immunofluorescent technique as follows. Sections were rinsed several times in PBS, followed by blocking in PBS containing 0.25% bovine serum albumin (BSA, Sigma) and 0.25% Triton-X 100 (Sigma) (PBS-TX-BSA) for 1 h at 4 °C. After that, sections were incubated for 24 h at 4 °C with different primary antibodies (Table 1) diluted in PBS-TX-BSA. Following washing in PBS-TX, the sections were incubated with a secondary antibody (Table 1) diluted in PBS-TX-BSA overnight at 4 °C, then mounted in a 3:1 mixture of glycerol and PBS. In case of double labeling, the sections were incubated for 24 h at 4 °C with the appropriate combination of the monoclonal anti-5-HT and one of the polyclonal primary antibodies. It was then followed first with an incubation with anti-mouse secondary antibody, then with a second incubation with anti-rabbit secondary antibody, each for 6 h at 4 °C. Finally, the sections were mounted in a 3:1 mixture of glycerol and PBS. The sections were viewed in a Leica TCS SP8 confocal laser scanning microscope (Leica Microsystems, Germany) equipped with appropriate wavelength-filter configuration settings. The necessary number of optical dections (15–30) with 0.5–0.8 μm step-size were made to capture of all visualized details. Image processing was performed by LasX (Leica Microsystems, Germany) software.

**Table 1:** Antibodies applied during the immunohistochemical procedure

Antibodies	Producer and catalog number; raised in; reference	Dilution
Primary antibodies		
Monoclonal		
Serotonin (5-HT)	Dako (Glostrup, Denmark) #Mo75801-2; mouse (Balog et al. 2012)	1:500
Polyclonal		
Histamine (HA)	Merck/Sigma (Darmstadt, Germany) #H7403; rabbit (Panula et al. 1990; Hegedűs et al. 2004; Baronio et al. 2018)	1:1000
Glutamate (Glu)	Merck/Sigma (Darmstadt, Germany) #AB5018; rabbit (Hatakeyama et al. 2007; Nivison-Smith et al. 2013)	1:1000
Tyrosine hydroxylase (TH)	Merck/Sigma (Darmstadt, Germany) #ab152; rabbit (Leksomboon et al. 2012; Lin et al. 2014)	1:500
FMRFamide (Fa)	ImmunoStar (Stillwater, MN) #20091; rabbit (Battonyai et al. 2018; Yurchenko et al. 2018)	1:1000
Mytilus inhibitory peptide (MIP)	Immunobiological Laboratories (Gunma, Japan) rabbit (Fujisawa 1996; Elekes et al. 2000)	1:1000
Secondary antibodies		
Fluorescein isothiocyanate (FITC)	Invitrogen (Carlsbad, CA) #A24507; donkey; anti-mouse	1:200
Tetramethylrhodamine (TRITC)	Invitrogen (Carlsbad, CA) #A16040; donkey; anti-rabbit	1:200

### HPLC–MS assay

#### Sample preparation

The transmitter (5-HT, DA, HA, Glu) content of different peripheral tissues (tentacle, lip, foot) and, for comparison, that of the whole CNS was measured. For extraction of signal molecules acetonitrile (50 µL/mg) was applied containing 0.1% formic acid and 0.01 m/v% dithiotreitol. Dopamine-1,1,2,2-d4 HBr (Sigma-Aldrich) internal standard was added to the tissues. The final concentration of internal standard was 100 ng/mL. Thereafter, tissues were homogenized and were explored with a high energy ultrasonicator UIS250V (Hielsher Ultrasound Technology) at 6 × 10 s, applying ice cooling between cycles. Samples were then vortex mixed and centrifuged (Heraeus Biofuge Pico, Thermo Fisher Scientific) at 10,000 rpm for 5 min. Spinning supernatants were loaded in pure tubes and the solvents were evaporated with a SpeedVac concentrator (Eppendorf Life Sciences) at room temperature. The samples were dissolved in 150 µL ultra-pure water containing 0.1% formic acid and loaded into autosampler vials for HPLC–MS measurements.

#### Measurements

Analyses were performed with a complex Ultimate 3000 (Dionex, Sunyvale, USA) micro HPLC system and a Qexactive UHR mass spectrometer (Thermo Fisher Scientific, Waltham, MA, USA). For the separations, gradient elution (Maasz et al. 2017) were performed on a security guard column equipped Kinetex PFP column (100 mm × 2.1 mm i.d., particle size 2.6 µm, Phenomenex, Torrance, USA). The mass spectrometer equipped with a HESI source was used in the positive ion mode for mass detection. Filters of parent ion scan (SIM-single ion monitoring) and fragment ion scan (MS/MS) modes were used for selective and sensitive detection of analytes. The most intense precursor-to-fragment transitions were used for quantitative analysis; for DA: 154.1 → 137.1 *m*/*z*, for 5-HT: 177.1 → 160.1 *m*/*z*, for HA: 111.1 → 95.1, for Glu: 148.1 → 84.1 and for d4-DA: 158.1 → 141.1. To the fragmentation 35 eV was applied as the normalized collision energy (Sarvari et al. 2014; Wei et al. 2014).

#### Quantification

Five-point calibration curves were made for the quantitative analysis, using 10.0; 50.0; 100.0; 500.0; 1000.0 pmol/ml monoamines and Glu as standards. Correlation coefficients (r2) were between 0.9674 and 0.9950 for all acceptable calibration curves both in parent ion scan (MS) and fragment ion scan (MS/MS) modes (not shown). The limit of detection and the limit of quantification were between 2.9–6.5 pmol/ml and 5.8–9.4 pmol/ml, respectively. The signal molecules were identified by their exact molecular weight and by their fragments (mentioned above) from the tissue homogenates. The quantification of data was made parallel both in MS and MS/MS modes.

## Results

### Immunohistochemical demonstration of signal molecules in the lip, tentacle and foot of *Lymnaea*

**Fig. 1:**
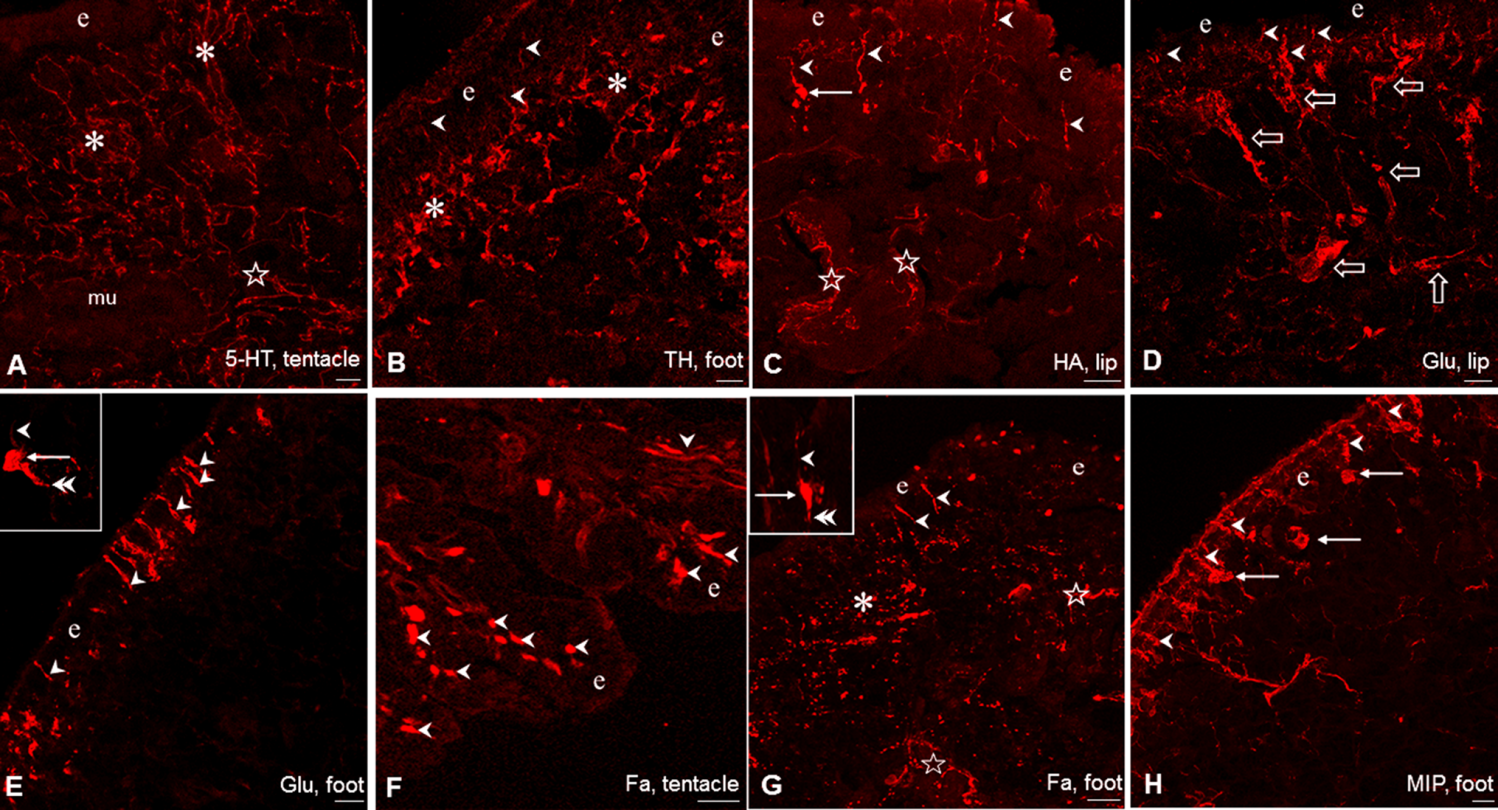
Immunohistochemical visualization of neuronal elements containing different signal molecules in the tentacle, lip and foot of *Lymnaea*. **A**, **B**, **G** Rich innervation of the sub-epithelial layer (asterisks) and deeper regions (stars) in the tentacle by 5-HT-IR (**A**), and by TH-IR (**B**) and Fa-IR (**G**) fibers in the foot. **C**–**F**, **H** Series of labeled sensory elements in the tentacle, lip and foot, showing both sensory perikarya (arrows) and sensory dendrite (arrowheads). In **C**, **G** stars mark labeled fibers in the deeper regions, and in **D** large open arrows indicate sensory axon bundles. Insets: enlarged view of a Glu- (**E**) and a Fa-IR (**G**) sensory neuron, respectively, in the foot with labeled perikaryon (arrow), dendrite (arrowhead), and axon process (double arrowhead). *e* epithelial layer. Scale bars: **A**–**C** 20 μm; **D**, **E**, **H** 30 μm; **F**, **G** 50 μm. Insets: **G** 10 μm; **E** 20 μm

Following the application of single labeling immunohistochemistry 5HT-IR, TH-IR, HA-IR, Glu-IR, Fa-IR and MIP-IR neuronal elements were present throughout the sensory region and subepithelial layer of the investigated peripheral organs, however, with partly different localization and density of occurrence (Fig. 1). 5-HT-immunoreactivity was bound exclusively to efferent axon processes forming a dense network at subepithelial level and below that in deeper regions (Fig. 1A). 5-HT-IR cell bodies did not occur at all.

**Fig. 2:**
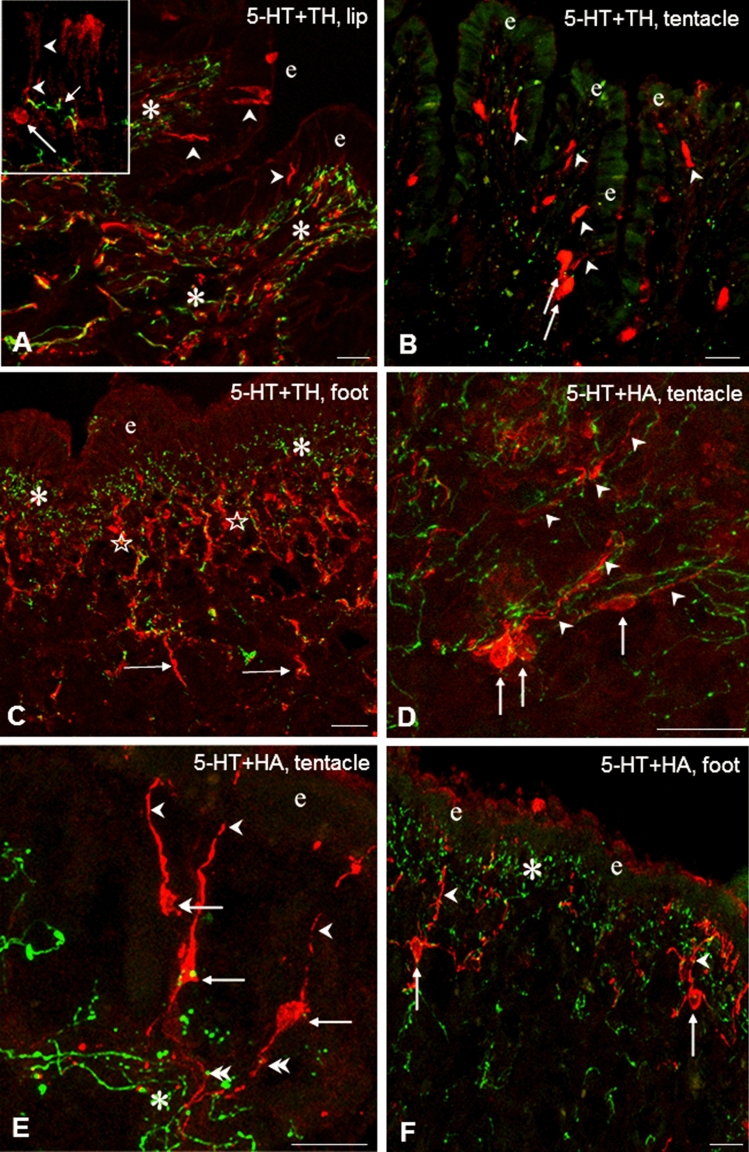
TH-IR (**A**–**C**, red) and HA-IR (**D**–**F**, red) sensory elements and their relationship to 5-HT-IR elements (green) in the tentacle, lip and foot of *Lymnaea*. **A** Innervation of a subepithelial segment in the lip by parallel running 5-HT-IR and TH-IR varicose fibers (asterisks). Note TH-IR sensory dendrites (arrowheads) in the epithelial layer (e). Inset: A TH-IR sensory neuron (arrow) projects with its dendrite (arrowhead) to the surface. Note a 5-HT-IR varicose fiber nearby (short arrow). **B** In the tentacle both TH-IR dendrites (arrowheads) and perikarya (arrows) are seen among scattered 5-HT-IR elements. e-epithelial layer. **C** Immense innervation of the subepithelial layer by 5-HT-IR (asterisks) and by perpendicularly running TH-IR (stars) fibers in the foot. Note TH-IR processes projecting further in the deeper region (arrows). **D** HA-IR sensory cells (arrows) with their dendrites (arrowheads) embedded in a network of varicose 5-HT-IR fibers (green) in the sub-epithelial layer of the tentacle. **E** HA-IR sensory neurons (arrow) displaying full morphology with dendrites (arrowheads) and axons (double arrowheads), the latter crossing 5-HT-IR varicose fibers. e-epithelial layer. **f** HA-IR sensory neurons (arrows) project with their dendrites (arrowheads) toward the surface of the epithelial layer (e) through the sub-epithelial 5HT-IR system (asterisk). Scale bars: **A**, **D**, **E**, **F** 20 μm; **B** 25 μm; **C** 30 μm. Inset: 10 μm

**Fig. 3:**
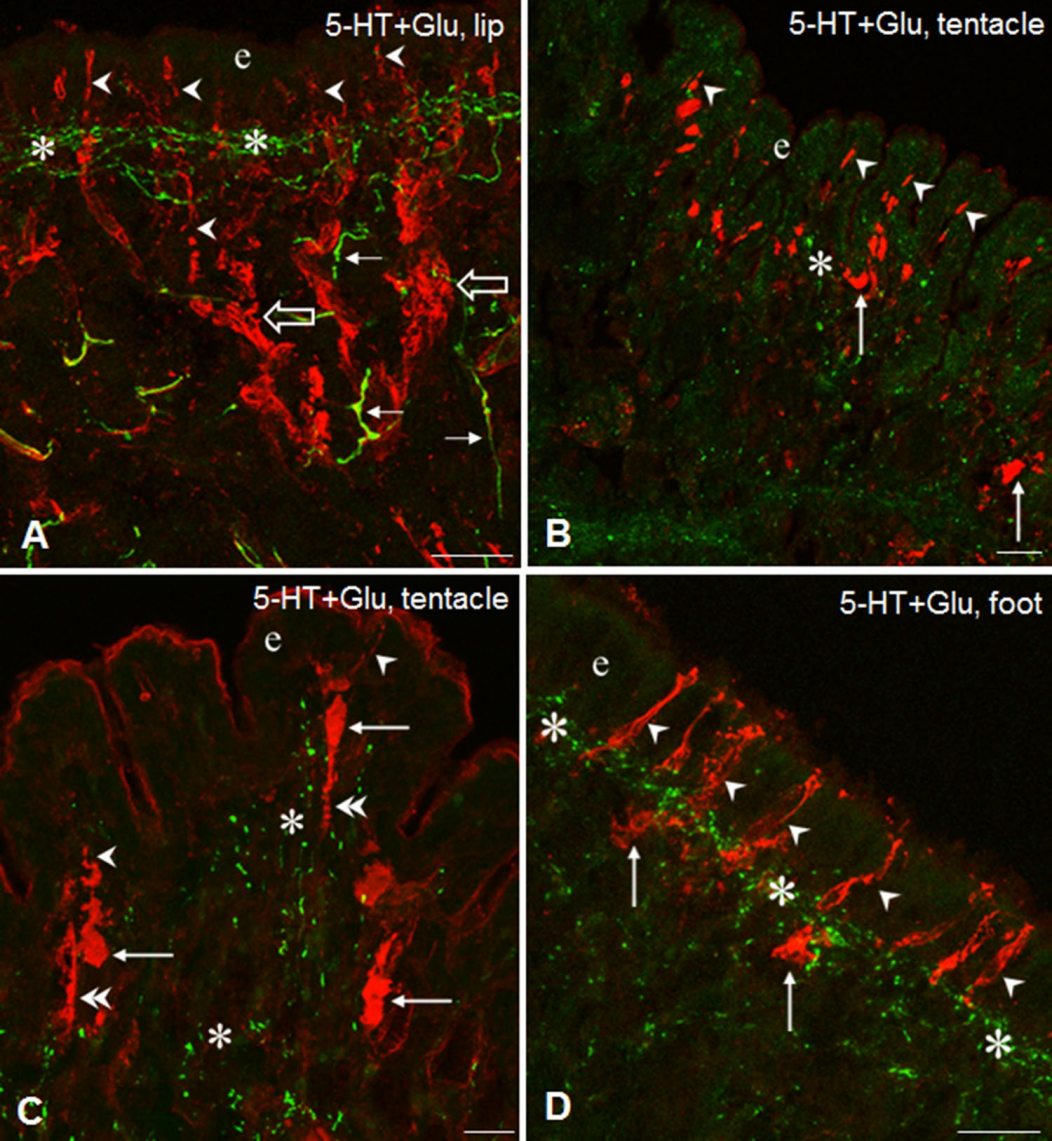
Glu-IR (red) sensory elements and their relationship to 5-HT-IR elements (green) in the tentacle, lip and foot of *Lymnaea*. **A** Glu-IR sensory dendrites (arrowheads) running to the surface of the epithelial layer (e) through the subepithelial 5-HT-IR network (asterisk) in the lip. Open arrows-thick Glu-IR sensory axon bundles, arrows- 5-HT-IR fibers. **B**, **C** Glu-IR sensory neurons in the tentacle with their sensory dendrites (arrowheads) in the epithelium (e), and perikarya (arrows) and sensory axons (double arrowheads) in the subepithelial layer (asterisk). **D** Series of Glu-IR sensory dendrites (arrowheads) running perpendicularly to the surface of the epithelium (e) across the 5-HT-IR subepithelial plexus (asterisks). Arrow-sensory perikarya. Scale bars: **A**, **D** 30 μm; **B** 40 μm; **C** 20 μm

**Fig. 4:**
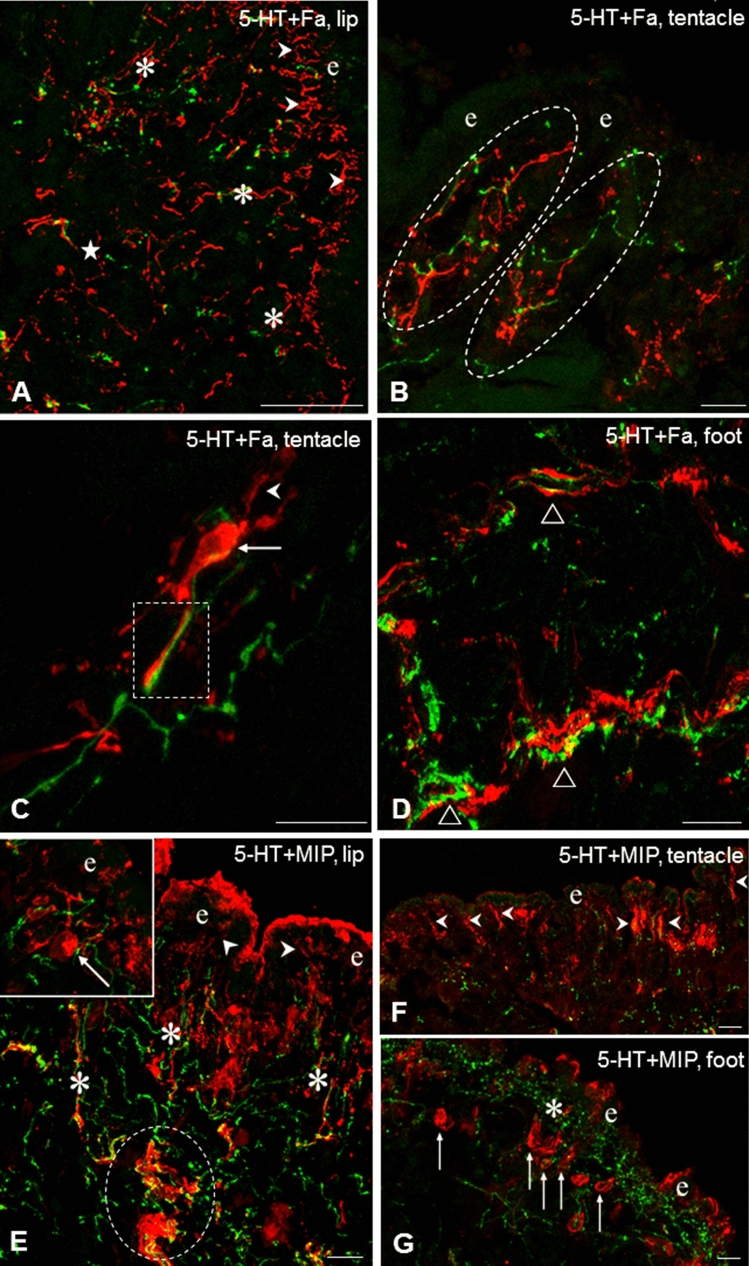
Fa-IR (Fa, **A**–**D**, red) and MIP-IR (**E**–**G**, red) sensory neurons and their relationship to 5-HT-IR elements (green) in the tentacle, lip and foot of *Lymnaea*. **A** Rich Fa-IR innervation both in the subepithelial (arrowheads) and deeper layers (asterisks) of the lip. Only scattered 5-HT-IR fibers can be seen. **B** Detail from the tentacle showing intermingled 5-HT-IR and Fa-IR varicose fibers (encircled) in the subepithelial layer. **C** Higher magnification of a long, interaction-like, close position (rectangle) of a Fa-IR sensory axon and a 5-HT-IR process in the tentacle. Arrow-the Fa-IR sensory cell body, arrowhead-sensory dendrite. **D** 5-HT-IR and Fa-IR axon processes running nearby (open triangles) in the sub-epithelial and deeper regions of the foot. **E** Localization of 5-HT-IR and MI-IR fibers in the subepithelial layer (asterisks) of the lip. Note 5-HT-IR varicose fibers located within and closely around a thick MIP-IR axon bundle (encircled). 5-HT-IR fibers running close around the sensory dendrite (arrowhead) and cell body (arrow) of a MIP-IR neuron. **F**, **G** MIP-IR sensory dendrites (**E**, arrowheads) in the lip and cell bodies (**F**, arrows) in the foot, accompanied with 5-HT-IR elements (asterisk). e-epithelial layer. Scale bars: **A** 50 μm; **B**, **C**, **E**, **F** 30 μm; **D**, **G** 20 μm

The localization of 5-HTergic neurons is restricted to the CNS in *Lymnaea stagnalis* (and other gastropod species as well). The cerebral giant cell (CGC) is responsible for the 5-HTergic innervation of the cephalic regions (see “Introduction”), whereas in case of the foot the 5-HT-containing efferent neurons are located in the pedal ganglia, although McKenzie et al. (1998) described the presence of a few 5-HT-IR cells in the foot. Other immunolabeled (TH-IR, HA-IR, Glu-IR, Fa-IR, MIP-IR) elements represented partly as bipolar sensory neurons, and partly as axon processes, forming either a subepithelial plexus or running in deeper regions beneath the plexus (Figs. 1B–H, 2C, 3A, 4A, E). In case of the lip and tentacle, the surface epithelium displayed a wavy form along certain segments, where the epithelial protrusions were supplied by parallel running immunolabeled fibers (Fig. 2A).

The sensory axons were collected first in smaller, later beneath the subepithelial layer in larger, thick bundles (Figs. 3A, 4E). However, whether the latter contained only afferent (sensory) elements or efferent fibers as well could only partly be determined unequivocally (see also below). The distribution pattern and organization of the IR sensory and other neuronal elements were similar in the lip and tentacle, meanwhile the picture in the foot showed certain differences. In the foot, TH-IR sensory neurons occurred less frequently, whereas a TH-IR subepithelial plexus and numerous TH-IR fibers could be observed beneath the plexus (Figs. 1B, 2C). Numerous Glu-IR and MIP-IR bipolar neurons were found in the anterior region of the foot, whereas only few HA- and Fa-IR sensory cells were present (Fig. 1E, G, H). Along certain surface segments perpendicularly running Glu-IR and MIP-IR dendrites were lined up closely near to each other (Figs. 1E, H, 3D, 4F). Both Fa-IR and MIP-IR subepithelial plexi, as well as Fa-IR and MIP-IR fiber networks could be observed in the deeper levels of the foot (Figs. 1G, 4G).

### Relationship of neuro-chemically different sensory (afferent) elements to 5-HTergic efferent innervation of central origin in the lip, tentacle and foot of *Lymnaea*

Double immunostaining was performed in the three peripheral organs to study the putative functional–anatomical relationship established between the sensory elements containing different signaling molecules and the 5-HT-IR efferent innervation. Following double labeling, a distinct, separated intracellular localization of the different immunolabelings could be observed. Co-localization of 5-HT-IR with other immunolabeled elements was not found. In the epithelial and subepithelial layers, efferent 5-HT-IR innervation was found in four different positions, related to the other five neurochemically different (TH-IR, HA-IR, Glu-IR, Fa-IR, MIP-IR) sensory structures. In all cases, the 5-HT-IR subepithelial plexus running parallel with the surface of sensory epithelium was found in close localization with different parts (dendrite, cell axon) of labeled sensory neurons (e.g. Figs. 2F, 3A, D, 4G**)**. In details, (1) the plexus was crossed perpendicularly by sensory dendrites (Figs. 2A, B, D, E, F, 3A, 4F); (2) a single 5-HT-IR fiber projected to a sensory dendrite in the outermost layer of the sensory epithelium (Figs. 2A, inset; 4E, inset); (3) labeled sensory cell bodies were embedded in the plexus (Fig. 4E, inset), and finally (4) 5-HT-IR and other IR fibers appeared together, mixed, either running parallel (Fig. 2A) or organized in an intimate network-like arrangement of varicose fibers at the wavy protrusions of the subepithelial region in the lip and tentacle (Fig. 4B). However, in these cases, the sensory and/or efferent character of the varicose fibers occurring together in close arrangement with the efferent 5-HT-IR axons could not be defined unequivocally. When certain segments of the epithelium was characterized by a wavy appearance in the tentacle and lip, 5-HT-IR and other immunolabeled processes invaded the subepithelial area of the protrusions (Fig. 2A). Finally, 5-HT-IR elements located in the deeper regions beneath the subepithelial layer appeared in various interaction-like arrangements with any of the other five immunolabeled systems (Figs. 2C, 4A D, E).

Close relationship between 5-HT-IR and other (sensory and non-sensory) IR structures could also be observed both in the subepithelial and deeper regions, suggesting interaction between these neuronal elements. Examples for sites of possible close intercellular contacts occurred in the tentacle, lip and foot sensory epithelial and subepithelial layers, where anatomically different parts (axon, cell body, dendrite) of sensory cells (Figs. 2D, E—HA-IR, 3A, D—Glu-IR, 4B, C—Fa-IR) were encompassed by 5-HT-IR efferent fibers bearing numerous varicosities. Also, in deeper regions, beneath the subepithelial layer, 5-HT-IR varicose fibers were found located over or running in close vicinity of other immunolabeled thick axon bundles (Fig. 4E).

### Concentration of signal molecules in peripheral organs (lip, tentacle and foot) of *Lymnaea*

**Fig. 5:**
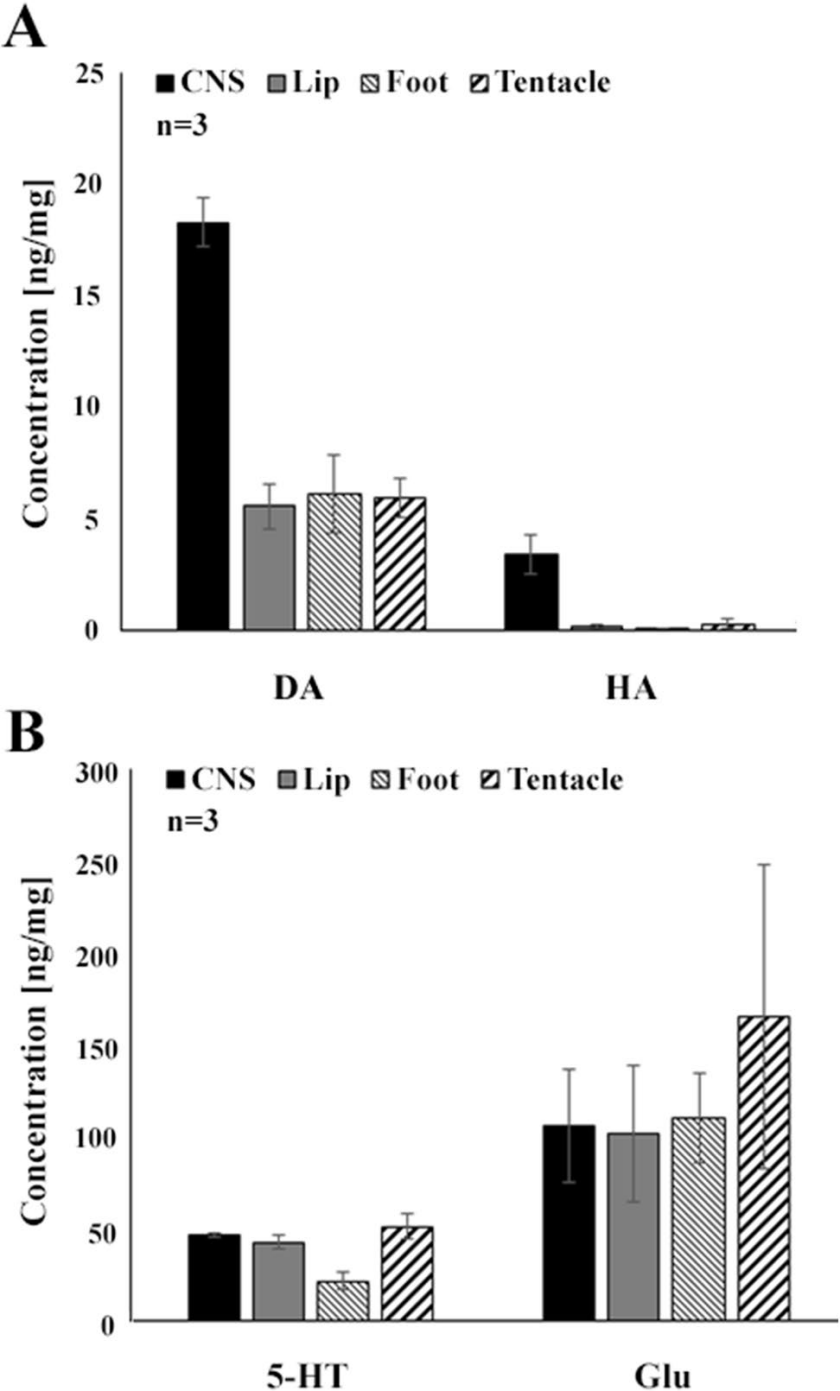
Monoamine and amino acid contents of peripheral organs (lip, tentacle, foot) of *Lymnaea*, determined by HPLC–MS assay. **A** Dopamine (DA) and histamine (HA), **B** serotonin (5-HT) and glutamate (Glu). Mean ± SD; *n* = 3

Associated with our immunohistochemical investigations, HPLC–MS measurements were carried out to identify and quantify the concentration of different signal molecules (5HT, DA, HA, Glu) in the foot, lip and tentacles. All the signal molecules assayed were detected in the peripheral organs, although, with markedly different concentrations (ng/mg) as follows (see also Fig. 5): Glu (lip—101,667; foot—110,136; tentacles—165,369;) > 5-HT (lip—42,683, foot—21,443, tentacles—51,222) > DA (lip—5568, foot—6095, tentacles—5971) > HA (0193—lip, foot—0082, tentacles—0297). As it can be seen, among the concentrations of Glu and 5-HT versus DA and HA there were orders of magnitude difference.

## Discussion

Our present results obtained on three different peripheral organs (lip, tentacle, foot) of the pond snail, *Lymnaea stagnalis*, indicate the following. (1) A broad range of (partly) still not identified signaling molecules is present in the neuronal elements of the epithelial and sub-epithelial area of these organs. (2) The signaling molecules visualized are possibly involved both in forwarding sensory information to the CNS and in the efferent innervation of the subepithelial and even deeper layers. (3) It is suggested that the subepithelial and deeper layers are the site of local processing, and modification of the sensory information by elements of central origin, although the primary site of interactions seems to be the subepithelial layer containing the plexus, (4) The subepithelial and deeper layers may also be the site of the innervation of muscular and glandular elements, as well as the source of 5-HTergic innervation of the ciliary cells, hence contributing to both exploring the source of the chemical and physical stimuli of the surrounding, and also elaborating responses to the environmental stimuli. (5) The lack of evidence for the co-localization of different transmitter substances refers to the separation of the neuro-chemically different signaling elements.

The presence of several signal molecules (DA [TH], HA, Glu, NO) in neuronal elements located in the sensory epithelium of different peripheral regions of adult and developing *Lymnaea* has been described earlier using fluorescence histochemistry and/or immunohistochemical staining. Glu containing bipolar sensory cells were described by Hatakeyama et al. (2007) and HAergic sensory elements were demonstrated by Hegedűs et al. (2004) in the lip and foot, whereas earlier and more recent data also indicated the presence of NO (NADPHd) in the lip, tentacle and foot (Serfőző et al. 1998; Wyeth and Croll 2011), and DA [TH], among others, in the foot, lip and tentacle (Croll et al. 1999; Wyeth and Croll 2011). In the current study, the presence of two additional signal molecules, the neuropetides, Fa and MIP, was demonstrated in sensory cells. Consequently, the pool of chemical substances involved in sensory signaling processes is wider than it has been known before (Wyeth and Croll 2011; Wyeth 2019). Analyzing the neuronal localization of the Fa gene encoded peptides (Benjamin and Burke 1994) in the developing, post-metamorphic *Lymnaea*, Voronehskaya and Elekes (2003) demonstrated sensory neurons in the lip, mantle and foot, displaying immunoreactivity to antibody raised against EFLRIamide, a member of the encoded peptide family, but no Fa-IR sensory neurons were found. At the same time, Fa-containing sensory cells were described in the tentacular olfactory system of other gastropod species like *Limax* (Suzuki et al. 1997) and *Aplysia* (Wollesen et al. 2007). In a preliminary study MIP-IR bipolar sensory cells were demonstrated in the mantle edge of late, post-metamorphic *Lymnaea* embryos (Elekes, unpublished), whereas in a detailed immunohistochemical study performed on adults only efferent MIP-IR fibers could be visualized in different peripheral regions (Elekes et al. 2000). Regarding other small molecular weight transmitters found in the *Lymnaea* periphery, HA and Glu were demonstrated in insect photoreceptor cells (Sarthy 2013; Nässel 2013).

Although qualitative immunohistochemstry cannot provide any information on the quantity of the signal molecules visualized in the periphery, certain consequences can though be drawn based on the results of our HPLC-assay. Out of the transmitters visualized both in the sensory elements and other neuronal components of the three peripheral organs studied, Glu was detected in the highest concentration, followed by DA. The high Glu concentration appear to be in good correlation with literature data, reporting that Glu is important neurotransmitter at the gastropod periphery (Walker 1986; Walker and Holden-Dye 1991; Kononenko and Zhukov 2005), whereas the relative high concentration of DA may correspond well to the frequent occurrence of TH-IR elements both in the sensory and lower subepithelial and deeper levels. It is also to be noted that out of the three peripheral regions, the highest concentration of the immunohistochemically visualized neurotransmitters, DA, HA and Glu, were detected in the tentacles, raising the role of these substances in transmitting sensory stimuli. The high concentration of 5-HT detected in the lip, tentacle and foot corresponds to the overall presence of the dense 5-HT-IR innervation. According to earlier literature data, there is a rich 5-HTergic projection system of central 5-HTergic neurons to the periphery, including the cephalic regions and the foot (Walker 1986; Syed et al. 1988; Walker and Holden-Dye 1991; Chase 2002; Balog et al. 2012). McKenzie et al. (1998) also detected significantly high 5-HT and DA concentrations in the *Lymnaea* foot and they correlated the presence of 5-HT with the demonstration of 5-HT-IR varicose fibers both under the epithelium and deeper regions. The ciliated epithelial cells of the *Lymnaea* foot were shown to stand under 5-HTergic regulation (Audesirk et al. 1979; Syed et al. 1988). The role of 5-HT in the early embryonic rotation perfomed by ciliary cells in *Lymnaea* was also demonstrated (Diefenbach et al.^1991^).

In a detailed IHC analysis, Wyeth and Croll (2011) demonstrated sensory cells containing HA, NO and catecholamines (DA), respectively, in the cephalic sensory organs (lip, tentacle) of *Lymnaea*. The study revealed that the three afferent signaling systems formed a network beneath the sensory epithelium, too. In our present study, it has been shown that both the subepithelial layer contains a dense 5-HT-IR nerve plexus running parallel with the surface epithelium, whereas another differently organized labeled network is located below. In case of double-labeling experiments, close, near-by localization of 5-HT-IR varicose elements, known to mark elements of exclusively extrinsic (central) origin, and labeled sensory axons could be observed. This anatomical arrangement raises a possible modulatory role of central (5-HTergic) input influencing the sensory information *en route*, before reaching the CNS. However, an inverse action cannot be excluded either, in which the sensory signal may exert a modulatory effect on elements of central origin. The morphological background of the possibility of the peripheral modulation has earlier been revealed for example in the snail (*Helix)* visceral nerve (Elekes et al. 1985) and the crayfish (*Orconecctes*) stretch receptors (Elekes and Florey 1987). In other marine gastropod species, e. g. *Aplysia, Tritonia, Pleurobranchea, Phestilla*, 5-HT-IR elements were shown to form glomerular structures in the the rhinophores (Moroz et al. 1997; Wertz et al. 2006; Wollesen et al. 2007; Faller et al. 2008), while in a detailed study on different peripheral tissues of *Pleurobranchea* and *Tritonia* Moroz et al. (1997) described both glomerular and transversally running organization of 5-HT-IR processes in sensory areas proposing for the latter a direct modulatory role of afferent pathways. At ultrastructural level, Gobbeler and Klussmann-Kolb (2007) have also demonstrated the presence of glomeruli in the cephalic sensory organs of different opistobranch species, responsible for processing the sensory information, meanwhile, like us, neither Zylstra (1972a, b) nor Zaitseva and Bocharova (1981) reported the presence of subepithelial glomerular structures or peripheral ganglia in *Lymnaea*.

In our present investigations, Fa-IR elements were also observed contributing to the subepithelial plexus, even if the density of this plexus was considerably lower compared to that established by 5-HT-IR fibers. The presence of Fa-containing axon processes and the role of Fa (and other oligopeptides) were demonstrated in the periphery of *Lymnaea* and closely related species (*Planorbis, Helisoma*) (Schot and Boer 1982; Sonetti et al. 1988; Bulloch et al. 1988; Buckett et al. 1990; Voronezhskaya and Elekes 2003 and see also Walker et al. 2009). In addition, Glu, HA and MIP are not to be excluded either to function as signal molecules in central efferents, forming connection with the sensory and other components (muscle fibers and gland cells) of the cephalic organs and the foot. Previous immunohistochemical studies delivered evidences that HA-IR, Glu-IR, and MIP-IR fibers, innervated several peripheral organs, including lip and foot of *Lymnaea* (Elekes et al. 2000; Hegedűs et al. 2004; Hatakeyama et al. 2007). Although not in *Lymnaea*, but in *Helix*, immunogold electron microscopic studies demonstrated close but unspecialized neuromuscular and neuro-glandular membrane contacts established by Fa-IR (Elekes and Ude 1994) and MIP-IR (Elekes 2000) axon varicosities. It seems that the efferent innervation of the subepithelial and deeper regions in the studied peripheral organs consists of a rich combination of signaling systems, supposedly involved in a complex form of local regulatory processes.

**Fig. 6:**
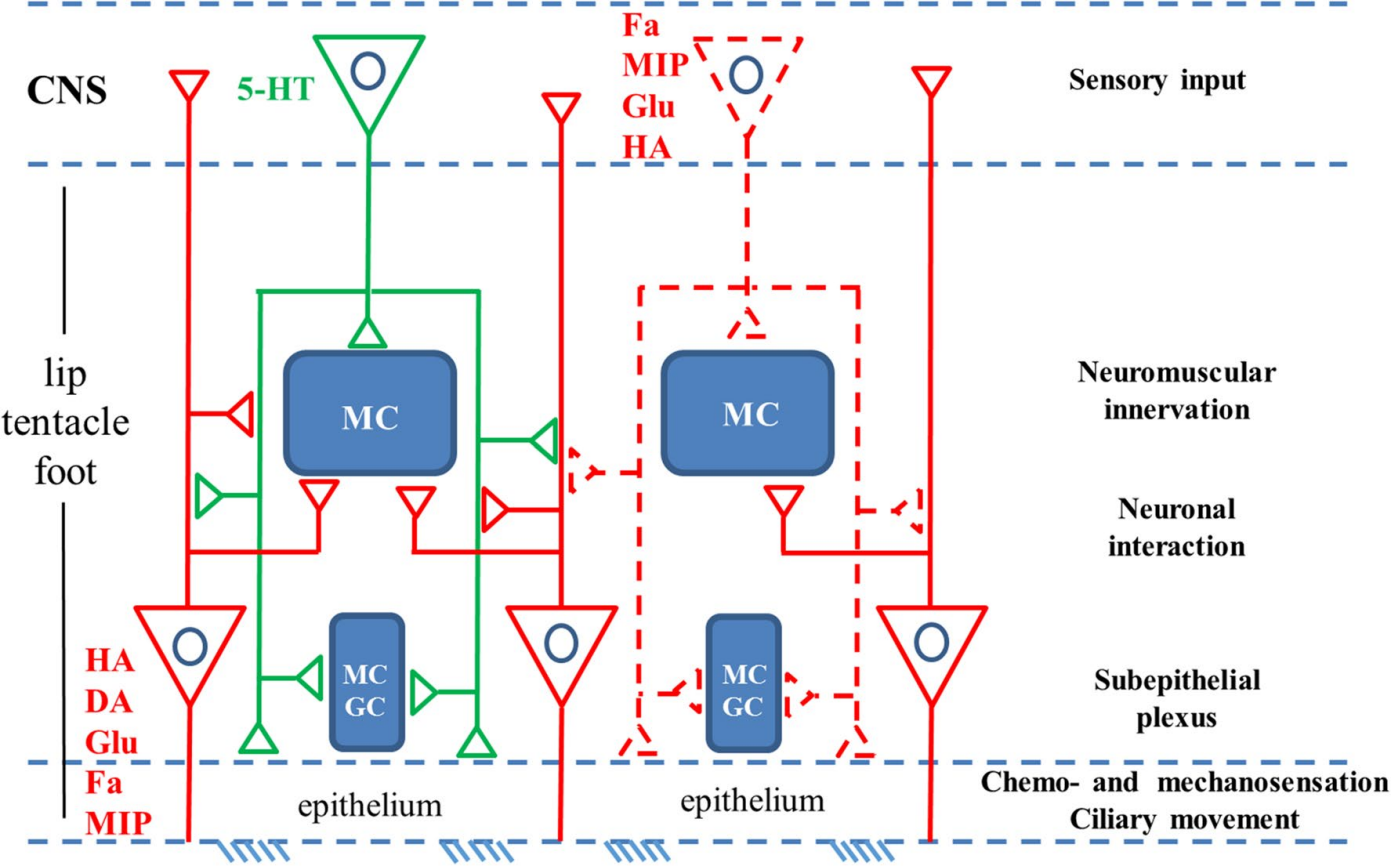
Schematic representation of levels and possible forms of innervation, as well as of the sensory-motor interaction in the lip, tentacle and foot of *Lymnaea*. Colors correspond to the immunofluorescence labeling (FITC-green, 5-HT, TRITC-red, others). Dashed red lines-putative (possible) innervation of central origin, in addition to 5-HT (based partly on literature data, see; Schot and Boer 1982; Walker 1992; Elekes et al. 2000; Hegedűs et al. 2004; Hatakeyama et al. 2007)

Recently, Wyeth (2019) has reviewed the neuronal (sensory-motor) background of the olfactory navigation in aquatic gastropods. Two ways of processing sensory information were suggested (see Fig. 7 in Wyeth 2019). One entering directly the CNS and another connected to a peripheral ganglion (or glomeruli) from where then, by inserting interneurons, efferent (motor) output is sent to the muscle system and the cilary epthelial surface as well. In contrast, based on our observations, summarized in Fig. 6, local, subepithelial and deeper networks seem to be potential sites of the sensory information processing in the tentacle, the lip and the foot of *Lymnaea*, without the involvement of a peripheral ganglion or subepithelial glomeruli. These types of local networks including interaction between the sensory and efferent elements may ensure fast and definitive responses to different sensory stimuli. In a recent study on *Lymnea*, Vehovszky et al. (2019) have demonstrated that following the application of the allelochemical tannic acid, both the afferent and efferent peripheral functions were affected. Feeding activity was reduced by blocking the sensory pathway and the locomotion activity was also inhibited, supposedly also through the sensory afferents. These observations may also refer to the important role of local (peripheral) elements in processing and/or modulating sensory information and the execution of appropriate efferent responses to various environmental cues. Peripheral circuits involved in sensory-motor prosesses were also suggested by Peretz et al. (1976) and Croll (2003) in case of the *Aplysia* gill withdrawal reflex. Croll (2003) also presented a scheme for the complex, central and peripheral innervation of the syphon and gill, in which two types of catecholaminergic neurons were demonstrated. One was a bipolar sensory cell and another a multipolar. Each projected to CNS motoneurons, but the multipolar cell ended also on the catecholaminergic sensory cells, hence forming a subepithelial modulatory level of the sensory information. The scheme, however, differs from that suggested by us in *Lymnaea*, in which no peripheral multipolar cells of any kind of signal molecule content was detected.
